# Sit or Squat? Toilet Type Is a Determinant of Diverticulosis Development

**DOI:** 10.5152/tjg.2024.23652

**Published:** 2024-06-01

**Authors:** Osman Çağın Buldukoğlu, Serkan Öcal, Galip Egemen Atar, Ferda Akbay Harmandar, Ayhan Hilmi Çekin

**Affiliations:** Department of Gastroenterology, Antalya Training and Research Hospital, Antalya, Türkiye

**Keywords:** Diverticulosis, colon, toilet type, sitting, squatting

## Abstract

**Background/Aims:**

Diverticulosis coli is a common disorder of the colon, and a luminal pressure increase in the colon is a proposed mechanism in disease pathogenesis. Toilet types used around the world can be fundamentally categorized into 2 categories: sitting toilets and squatting toilets. Squatting toilets are shown to lead to better puborectalis muscle relaxation, wider anorectal angle, and require less straining compared to sitting toilets. Stemming from this knowledge, we hypothesized that toilet type would play a role in the complex pathogenesis of diverticulosis and that squatting toilets would lower the risk of diverticula formation.

**Materials and Methods:**

This study was conducted at Antalya Training and Research Hospital between January 2023 and July 2023. A 1-page questionnaire consisting of demographic data and bowel habits as well as diverticulosis-related parameters was prepared to gather the study data. Colonoscopy results were matched with corresponding questionnaires.

**Results:**

The study population consisted of 929 patients. Advanced age was found to be a risk factor for diverticulosis. Sitting toilet was also found to be a risk factor for diverticulosis in multivariate logistic regression analysis with an odds ratio of 3.36 (95% CI: 1.684-6.705) (*P* = .001).

**Conclusion:**

The results of this study revealed that toilet type is a determining factor in diverticulosis development, as was hypothesized during the conceptualization of the study. Revealing the potential contribution of the toilet type used to the development of a relatively common and impactful disorder like diverticulosis will lay the bedrock for future studies on the topic.

**Main Points**

- Diverticulosis coli is a common disorder with a complex pathogenesis and high colonic intraluminal pressure plays a role in diverticula formation.
- Sitting toilets require more straining hence increased intra-abdominal pressure during defecation compared to squatting toilets. Anatomical differences during the defecation process between sitting and squatting toilets also contribute to the degree of pressure required to expel feces.

- This study revealed that toilet type plays a role in diverticula formation. Sitting toilet is found to be a risk factor for diverticulosis coli, which is an impactful finding for both the lifestyle of the population and future studies on the subject.

## Introduction

Diverticulosis coli is a common and burdensome disease of the colon with a varying clinical picture of silent disease to severe inflammation and bleeding. The underlying pathology is complex and not fully understood although there are some established and proposed risk factors. Genetics, age, inflammation and lifestyle all play roles in disease pathogenesis.^1^ Luminal pressure increase in the colon secondary to straining during defecation is also related to diverticulosis.^2^

Defecation is a complex physiological function which involves the gastrointestinal, nervous, and musculoskeletal systems and requires these systems to work in coordination in order to successfully expel the feces.^3^ Contraction of the abdominal muscles along with relaxation of the external anal sphincter and puborectalis muscle will lead to the expulsion of feces due to the pressure gradient between the anal canal and the rectum.^4^

Different toilet types are used around the world, but fundamentally they can be categorized into 2 main headings: sitting toilets (referred to as western toilet, European toilet, “alla franca” toilet, or pedestal latrine) and squatting (squat toilet, “alla turca” toilet) toilets. Squatting toilets are shown to lead to better puborectalis muscle relaxation, a wider anorectal angle (ARA), and require less straining, all of which lead to an easier, prompt, and complete fecal evacuation compared to sitting toilets.^5,6^ Less straining translates to a relatively lower increase in abdominal pressure during defecation in a squatting position compared to sitting.

Current literature lacks studies investigating the toilet types and their contribution to the development of diverticulosis. Stemming from this knowledge, we propose that toilet type would play a role in the complex pathogenesis of diverticulosis, and squat toilets would lower the risk of diverticula formation.

## Materials and Methods

### Patients

This study was conducted at Antalya Training and Research Hospital, Türkiye, over a span of 6 months between January 2023 and July 2023. A 1-page questionnaire was prepared to gather the data on the demographics of the patient population and factors that would likely affect diverticulosis presence. The questionnaire consisted of the following questions: age, sex, weight, height, smoking status, alcohol consumption, regular exercise (at least 3 times a week and at least for 30 minutes each session), sufficient water consumption (at least 2.5 liters per day), acetylsalicylic acid (ASA) usage (regularly), non-steroidal anti-inflammatory drug (NSAID) usage, presence of constipation (as per Rome IV criteria), need to strain during defecation, and the type of toilet used. A visual Bristol Stool Form Scale (BSFS) was included in the questionnaire, and patients were asked to mark their most common and prominent form of stool from the chart. Bristol Stool Form Scale scores 3 and 4 were considered normal. Scores 1 and 2 were considered on the constipation side of the spectrum, whereas 5, 6, and 7 were considered on the diarrhea side.

Patients who attended the gastroenterology endoscopy unit to undergo colonoscopy were included in the study. Questionnaires were handed out and collected from the patients before the procedure. Colonoscopy results of patients were matched with corresponding questionnaires at the end of each day. Only patients above 18 years of age were included in the study. Patients who could not answer the questionnaire for any reason and patients undergoing urgent colonoscopy were not included in the study.

### Statistical Analysis

Continuous data were represented as mean ± standard deviation or median with percentiles (25-75 percentiles) on the basis of the distribution, which was controlled by Shapiro–Wilk test. Categorical variables were given with frequencies (n) and percentages (%). Pearson chi-square, Yates’ chi-square test, and Fisher’s Exact test were used to determine the relationship between categorical variables. For normally distributed data, comparisons between 2 groups were analyzed by independent *t*-test, while more groups by one-way analysis of variance and multiple comparison test (Tukey). Mann–Whitney *U*-test and Kruskal–Wallis test was performed for non-parametric comparisons of continuous data. Post hoc analysis was performed using Bonferroni correction. The optimal cutoff value of age differentiating diverticulosis was evaluated with the receiver operating characteristic curve analysis. The area under the curve, sensitivity, and specificity were calculated and reported with 95% CIs. Multivariate logistic regression analysis was used to establish which predictor variables were significantly related to the divertikul. Odds ratio (OR) with corresponding 95% confidence intervals (95% CIs) were reported. All statistical analyses were carried out using IBM SPSS Statistics for Windows, version 23.0 (IBM Corp., Armonk, NY, USA). A value of 2-sided *P* < .05 was considered statistically significant.

### Ethics Committee Approval

Ethics committee approval for the study was obtained from University of Health Sciences, Antalya Training and Research Hospital Ethics Committee (number: 2022/361, date: 22/12/2022). Written informed consent was obtained from each participant in the study for participation in the study.

## Results

### Characteristics of the Study Population and Their Relationship with Diverticulosis

A total of 929 patients were enrolled in the study. The average age of the study population was 56.47 ± 13.67 years. The male to female ratio was close to 1 : 1. Of the patients in the study, 59.9% (n = 557) were overweight (body mass index >25 kg/m^2^). Almost half of the study population needed to strain during defecation (48.4%), and 99 patients (10.7%) had constipation. A total of 304 patients were using squatting type toilets (32.7%), whereas 625 patients were using sitting type toilets (67.3%). Of the patients, 486 (52.3%) had normal BSFS scores. Diverticulosis was diagnosed in 81 patients (8.7%). Patient characteristics of the study population are given in Table 1.

Age was found to be related to diverticulosis, and this relationship was statistically significant (*P* < .001). Mean age of patients with diverticulosis was 64.46 ± 8.94 years, compared to 55.71 ± 13.81 years of mean age in patients without diverticulosis. A relationship was found between ASA usage and diverticulosis, with 34.6% of patients with diverticulosis using ASA regularly compared to 20.9% of patients without diverticulosis (*P* = .007). Sitting toilets were found to be used more commonly in patients with diverticulosis. Ten patients with diverticulosis (12.3%) were using squatting toilets, and 71 of patients with diverticulosis were using sitting toilets (87.7%), and this difference was statistically significant (*P *< .001). Location of colonic diverticula was not related to the type of toilet type used. Other study parameters were not found to be correlated with the presence of diverticulosis. The relationship of patient characteristics with diverticulosis is given in Table 2.

Patients were categorized according to their BSFS scores and analyzed with study parameters. Patients with a higher BSFS score^5-7^ were found to be younger compared to patients with BSFS scores of 1 to 4, and this difference was statistically significant (*P* = .038). Female patients had lower BSFS scores compared to males. A total of 110 patients (60.8%) were female in the BSFS score 1-2 group compared to 71 (39.2%) male patients, and this difference was statistically significant (*P* = .003). Expectedly, patients with constipation had low BSFS scores and 30.4% of patients with BSFS scores of 1-2 were constipated. This percentage was 6.6% for BSFS scores 3-4 and 4.6% for BSFS scores of 5-7. This difference between the patient groups according to BSFS scores was statistically significant (*P* < .001). Straining was also more common in the BSFS score 1-2 group compared to patients with a BSFS score of 3 or higher, with 134 patients (74%), and this finding was statistically significant (*P* < .001). The results are given in Table 3.

### Analysis of Study Parameters with Diverticulosis Development

Multivariate logistic regression analysis was performed to determine the factors associated with diverticulosis. The analysis revealed that older age and sitting toilet are independent risk factors for diverticulosis. Age had an OR of 1.051 (*P* < .001) whereas sitting toilet had an OR of 3.36 (*P* = .001). Results are given in Table 4.

Patients were subclassified into 2 groups by each toilet type and 3 groups by BSFS scores (as scores of 1-2, 3-4, and 5-7) and their relationships with diverticulosis were analyzed. Out of 81 patients with diverticulosis, 51 patients (62.9%) were in the BSFS 3-4 group, which was designated as the normal BSFS group. In this subgroup of 51 patients with diverticulosis, 6 patients (11.8%) were using squatting toilets compared to 45 patients (88.2%) using sitting toilets, and this difference was statistically significant (*P* = .002). Results are given in Table 5.

## Discussion

The study was based on the hypothesis that since squatting during defecation leads to better puborectalis muscle relaxation, a wider ARA, and requires less strain compared to sitting; sitting toilets would be a risk factor for diverticulosis development owing to the increased intra-abdominal pressure required for defecation. The results revealed that sitting toilets are indeed linked to a higher rate of diverticulosis compared to squatting toilets.

Human defecation is a complex physiological function which requires multiple organ systems to work in coordination and harmony. Musculoskeletal system, neurologic system, gastrointestinal system, and mental wellbeing of the patient all play roles in the successful excretion of feces.^7,8^ Pelvic floor muscles, mainly the puborectalis muscle, play an important role in the achievement of a wide rectoanal angle during defecation. Anal sphincter function is another component of defecation and the external anal sphincter is also shown to be affected by puborectalis muscle.^9-11^ Anorectal angle is the angle between the long axis of the anal canal and the posterior rectal line. While resting, the physiologic range of ARA is between 65 to 100 degrees. Puborectalis muscle contraction leads to a more acute ARA, which is crucial in maintaining fecal continence. On the other hand, relaxation of the puborectalis muscle leads to a wider ARA, leading to evacuation of feces.^12,13^ Disturbance in one or more of these mechanisms leads to dyssynergic defecation, which is an important cause of chronic constipation.^14^ Squatting during defecation leads to a more relaxed puborectalis muscle and a wider ARA, thus leading to a decrease in the need of straining and a smoother and more complete feces excretion compared to sitting.^5,15,16^ The culmination of all the aforementioned data is a more physiologic defecation posture in the squatting position.

The earliest toilet structures in history were found in the Indus basin, dating back to 3000 BC.^17^ Over the course of history, communal toilets were built especially in big cities with Rome being famous for having a sewer system around 2000 years ago. A vast majority of toilets were squatting toilets although rare findings of sitting toilets were also seen.^18^ Since then, sitting and flushing toilets became more popular, especially in the ruling class. The last few centuries saw a great shift from squatting toilets to sitting toilets globally, where sitting toilets are almost uniformly used in the western world.

In light of the given data, a study comparing the 2 types of toilets—which are both commonly used in Türkiye—in terms of their relationship with diverticulosis was planned.

As expected by our hypothesis, sitting toilet was found to be a risk factor for diverticulosis in multivariate logistic regression analysis with an odds ratio of 3.36 (95% CI: 1.684-6.705) (*P* = .001). A similar study by Ozturk et al^19^ in 2018 revealed similar results, with the usage of sitting toilets higher in patients with diverticulosis. As mentioned before, this elevated risk can be owed to the increased need of straining secondary to defecation posture and related anatomic status ending in a rise in the intra-abdominal pressure which is thought to partake in the pathogenesis of diverticulosis. The study also revealed age to be a risk factor for diverticulosis, expectedly as per literature.^20,21^

Evaluation of BSFS scores was also added to the study with the chart being added to the study questionnaire. The rationale was since patients with loose stools (BSFS 5-7) would have a faster defecation with less straining requirements, abdominal pressure would be lower than patients with lower BSFS scores leading to a lower rate of diverticulosis. Although the study results revealed that patients with higher BSFS scores had less need to strain during defecation, there was no relationship between BSFS scores and the presence of diverticulosis. Female patients were found to be more common in the BSFS score 1-2 group than male patients.

Role of toilet types on disorders like diverticulosis, irritable bowel syndrome, and hemorrhoids has been debated in the literature; but most studies are at a theoretical level and lack large population based studies with cause and effect relationships.^22-24^ A practice guideline published by Indian Motility and Functional Diseases Association and the Indian Society of Gastroenterology also commented on the topic. Squatting toilets were stated to be more physiological than sitting toilets for defecation in the consensus report.^25^ There are studies parallel to these findings to alleviate the negative effects of sitting toilets to better mimic the defacation physiology. Using a footstool to achive a wider ARA during defecation while sitting was found to be efficient.^26^ There are also squat-assist devices being developed especially for elderly patients with defecation problems.^27^

There are some limitations of the study. First of all, even though the study population is adequate for analysis, a higher number of patients with diverticulosis would be preferable to better establish a link between toilet type and disease pathogenesis. A cohort study comparing 2 populations using different types of toilet and assessing development of diverticulosis over time would be optimal. Patients prefer to use sitting toilets as they age so whether or not they changed their toilet type during their lifetime would have added more data for the study. Second, more data on defecation patterns such as time spent during defecation, presence of sense of complete defecation and presence of other defecation-related problems would have been valuable. Acetylsalicylic acid dosage and duration of use, as well as comorbidities and dietary habits of the study population would have added valuable information for further analysis. Last, incorporation of manometric measurements would give valuable data to back up the study findings.

Overall, the results of this prospective study with a relatively large number of patients revealed important and valuable information on the relationship between toilet types and diverticulosis development. Based on this data, future studies focusing on not only diverticulosis but also other bowel disorders and their relationship with the type of toilets used can impact social life and possibly open a new perspective on public health.

## Figures and Tables

**Table 1.** Patient Characteristics of the Study Population.

Variables	Patients (n = 929)
Age (years), mean *± *SD	56.47 ± 13.67
Sex, n (%)	
Male	464 (49.9)
Female	465 (50.1)
BMI, median	26.3
BMI group, n (%)	
≤ 25	372 (40)
> 25	557 (59.9)
Smoking, n (%)	228 (24.5)
Alcohol, n (%)	32 (3.4)
Exercise, n (%)	406 (43.7)
Water intake, n (%)	672 (72.3)
ASA usage, n (%)	205 (22.1)
NSAID usage, n (%)	144 (15.5)
Constipation, n (%)	
No	830 (89.3)
Yes	99 (10.7)
Toilet type, n (%)	
Squatting	304 (32.7)
Sitting	625 (67.3)
Straining, n (%)	450 (48.4)
BSFS, *median*	4
BSFS groups, n (%)	
1-2	181 (19.5)
3-4	486 (52.3)
5-7	262 (28.2)
Diverticulosis, n (%)	81 (8.7)
Diverticulosis location	
Left-sided	68 (83.9)
Right-sided	4 (4.9)
Pan-colonic	9 (11.1)

ASA, acetylsalicylic acid; BMI, body mass index; BSFS, Bristol Stool Form Scale; NSAID, non-steroid anti-inflammatory drug; SD, standard

**Table 2.** Relationship of Patient Characteristics with Diverticulosis.

Variables	Diverticulosis Not Present (n = 848)	Diverticulosis Present (n = 81)	*P*
*Age (years), mean ± SD*	55.71 ± 13.81	64.46 ± 8.94	<.001
Sex, n (%)			
Male	416 (49.1)	48 (59.3)	.079
Female	432 (50.9)	33 (40.7)	
BMI, median	26.2	27.1	.395
BMI group, n (%)			
≤ 25	342 (40.3)	30 (37.0)	.694
> 25	506 (59.6)	51 (62.9)	
Smoking, n (%)	215 (25.4)	13 (16)	.085
Alcohol, n (%)	28 (3.3)	4 (4.9)	.516
Exercise, n (%)	367 (43.3)	39 (48.1)	.399
Water intake, n (%)	612 (72.2)	60 (74.1)	.813
ASA usage, n (%)	177 (20.9)	28 (34.6)	.007
NSAID usage, n (%)	130 (15.3)	14 (17.3)	.761
Constipation, n (%)			
No	758 (89.4)	72 (88.9)	.999
Yes	90 (10.6)	9 (11.1)	
Toilet type, n (%)			
Squatting	294 (34.7)	10 (12.3)	<.001
Sitting	554 (65.3)	71 (87.7)	
Straining, n (%)	411 (48.5)	39 (48.1)	.956
BSFS, *median*	4	4	.907
BSFS groups, n (%)			
1-2	170 (20)	11 (13.6)	.115
3-4	435 (51.3)	51 (63)	
5-7	243 (28.7)	19 (23.5)	

ASA, acetylsalicylic acid; BMI, body mass index; BSFS, Bristol Stool Form Scale; NSAID, non-steroid anti-inflammatory drug; SD, standard deviation.

**Table 3.** Relationship of Bristol Stool Form Scale Scores with Study Parameters

Variables	BSFS 1-2 (n = 181)	BSFS 3-4 (n = 486)	BSFS 5-7 (n = 262)	*P*
Age (years), mean *± *SD	57.56 ± 13.21^a^	57.03 ± 13.09^a^	54.67 ± 14.89^b^	.038
Sex, n (%)				
Male	71 (39.2)^a^	262 (53.9)^b^	131 (50)^b^	.003
Female	110 (60.8)^a^	224 (46.1)^b^	131(50)^b^	
Smoking, n (%)	38 (21)	127 (26.1)	63 (24)	.381
Alcohol, n (%)	3 (1.7)	20 (4.1)	9 (3.4)	.302
Exercise, n (%)	80 (44.2)	200 (41.2)	126 (48.1)	.187
Water intake, n (%)	141 (77.9)	348 (71.6)	183 (69.8)	.154
ASA usage, n (%)	45 (24.9)	108 (22.2)	52 (19.8)	.454
NSAID usage, n (%)	32 (17.7)	74 (15.2)	38 (14.5)	.648
Constipation, n (%)				
No	126 (69.6)^a^	454 (93.4)^b^	250 (95.4)^b^	<.001
Yes	55 (30.4)^a^	32 (6.6)^b^	12 (4.6)^b^	
Toilet type, n (%)				
Squatting	65 (35.9)	154 (31.7)	85 (32.4)	.582
Sitting	116 (64.1)	332 (68.3)	177 (67.6)	
Straining, n (%)	134 (74)^a^	225 (46.3)^b^	91 (34.7)^c^	<.001

One-way analysis of variance, Kruskal–Wallis test, Pearson chi-square test. Same letters in a row denote the lack of statistically significant difference.

ASA, acetylsalicylic acid; BSFS, Bristol Tool Form Scale; NSAID, non-steroid anti-inflammatory drug; SD, standard deviation.

**Table 4.** Factors Associated with Diverticulosis

Variables	OR (95% CI)	*P*
Age	1.051 (1.027-1.075)	<.001
Male gender	1.584 (0.977-2.568)	.062
Smoking	0.746 (0.392-1.42)	.373
ASA	1.155 (0.684-1.95)	.589
Sitting toilet	3.36 (1.684-6.705)	.001

Multivariate logistic regression analysis.

ASA, acetylsalicylic acid.

**Table 5.** Relationship of Toilet Types and Bristol Stool Form Scale Scores with Diverticulosis

BSFS groups, n (%)	Squatting	Sitting	*P*
BSFS 1-2			
Diverticulosis absent	64 (98.5)	106 (91.4)	.100
Diverticulosis present	1 (1.5)	10 (8.6)	
BSFS 3-4			
Diverticulosis absent	148 (96.1)	287 (86.4)	.002
Diverticulosis present	6 (3.9)	45 (13.6)	
BSFS 5-7			
Diverticulosis absent	82 (96.5)	161 (91)	.175
Diverticulosis present	3 (3.5)	16 (9)	

Yates’ chi-square test, Fisher’s exact test.

BSFS, Bristol Stool Form Scale.
